# Maternal undernutrition as proxy indicators of their offspring’s undernutrition: evidence from 2011 Ethiopia demographic and health survey

**DOI:** 10.1186/s40795-019-0281-z

**Published:** 2019-03-01

**Authors:** Alinoor Mohamed Farah, Bilal Shikur Endris, Seifu Hagos Gebreyesus

**Affiliations:** 1 Department of Public Health, College of Medicine and Health Sciences, Jigjiga University, Jigjiga, Ethiopia; 2 Department of Reproductive Health and Health Service Management, School of Public Health, College of Health Sciences, Addis Ababa University, Addis Ababa, Ethiopia

**Keywords:** Child, Maternal, DHS, Ethiopia, Undernutrition, BMI, Height

## Abstract

**Background:**

The intergenerational continuity of undernutrition is influenced by shared genetic, household socio-economic and cultural resources which will be associated with the mother and the child nutritional status, possibly to the same degree. Provided that this assumption is valid, maternal height and BMI could be a simple way of measuring nutritional status of their children.

**Methods:**

Data were obtained from the 2011 Ethiopia Demographic and Health Survey (EDHS 2011). An analytical sample of 8, 505 children whose mothers are not pregnant and live with their biological mothers was included. The bivariate associations between nutritional indices of the mother and the children were analyzed with Pearson correlation coefficients. The sensitivity, specificity, predictive values and area under Roc curves were calculated. Logistics regression for binary outcomes was also used to evaluate the predictors of child undernutrition.

**Results:**

Children who experienced stunting, underweight or wasting had mothers with lower mean BMI than those who did not (*p* < 0.001). Maternal and child nutritional status were positively correlated. The sensitivity of maternal underweight (defined by BMI < 18.5 kg/m^2^) as a predictor of child’s nutritional status (<− 2 z-scores) is low, failing to reach 50% for any of the child nutrition indices. In logistics regression, maternal BMI was associated with stunting, underweight and wasting (*p* < 0.001) while maternal height was only associated with stunting and underweight (*p* < 0.001).

**Conclusion:**

The sensitivity and specificity of maternal anthropometric indicators to identify growth deficits among children were too low to justify using maternal indicators as a replacement for child growth measurements.

## Background

Anthropometry is widely used tool to assess the nutritional status of children and to monitor their growth [1]. Further, the prevalence of undernutrition in children particularly those aged 6–59 month is used as an indicator for nutritional status of the entire population since they are more sensitive to nutrition related stress [2].

A number of anthropometric indicators have been used assessing nutritional status of children and they include; weight-for-height, weight-for-age and height for age, among others. Multicomponent indicators such as weight for age and height for age require age to be determined accurately since they are more sensitive to random errors in age than in anthropometry [3]. Therefore, in a setting where vital statistics are not recorded or exact ages are not known it will be difficult to assess accurately the nutrition status of children.

Likewise, younger children are more difficult to deal with during weight and height measurements and result in error [4]. In other words, at least two persons are required to measure weight and height accurately: one to take measurements and other to record [5]. In situations where there are no sufficient health workers it may be also difficult to undertake these measurements accurately.

Considering the intergenerational continuity of undernutrition, maternal BMI and height which are independent of age and relatively easier to measure could be a simple way of measuring nutritional status of their children and as a result they can be potential proxy indicators for nutritional status of the entire population.

Therefore, the current study is aimed to determine whether maternal BMI and height can be used to assess child undernutrition and to explore the predictors of child undernutrition by analyzing a heterogeneous study population from a nationally representative sample.

## Methods

### Study setting

Ethiopia is a country with 94 million people, second largest among African countries and among the least urbanized countries in the world. The majority of the population resides in the highland areas [6]. The source of livelihood of the settled rural population is farming while the lowland areas are mostly inhabited by nomads, who depend mainly on livestock production and move from place to place in search of pasture and water.

There are 11 administrative regions in Ethiopia (9 regional states and two administrative cities); Tigray, Afar, Amhara, Oromia, Somali, Benishangul-Gumuz, Southern Nations Nationalities and Peoples (SNNP), Gambella, Harari, Addis Ababa, and Dire Dawa. Regions are divided into zones, and zones, into administrative units called *woredas*. Each *woreda* is further subdivided into the lowest administrative unit, called *kebele*. More than 80% of the country’s total population lives in the regional states of Amhara, Oromiya, and SNNP.

The 2011 EDHS is the third Demographic and Health Survey conducted in Ethiopia. It is intended to measure levels, patterns, and trends in demographic and health indicators. EDHS provides data on fertility, family planning, maternal and child health, childhood mortality, nutrition, malaria, HIV knowledge and behavior, and HIV prevalence [6].

### Data source

Secondary analysis was performed using data from the 2011 Ethiopian Demographic Health Survey (EDHS), which is a nationally representative cross-sectional household survey of women of reproductive age and children less than five years old in Ethiopia. The data have been weighted to cater for the different sample proportions [6]. The survey was conducted from September 2010 to January 2011 and included three structured questionnaires: the Household Questionnaire, the Woman’s Questionnaire, and the Man’s Questionnaire.

### EDHS sample design and procedure

A representative probability sample of 18,720 households was selected using a multistage stratified two- stage cluster sampling design in which samples of households within clusters (enumeration areas) are selected. This sample was constructed to allow for separate estimates of health indicators for each of the 11 geographic/administrative regions (nine regional states and two city administrations), as well as for urban and rural areas separately. A total of 624 clusters, 187 urban and 437 rural were selected from the sampling frame (The 2007 Population and Housing Census) in the first stage. In the second stage, a fixed number of 30 households were selected for each enumeration area. Of all the selected 18,720 households, 5610 are in urban areas and 13,110 are in rural areas [6].

### Analytic sample and population

The EDHS sample design considers different parameters for the indicators to estimate the final sample size. In view of that, we have used children recode file with 7764 women and 11,654 children. But our study focused only on children whose mothers are not pregnant and living with their biological mothers.

### Study variables

#### Outcome variables

Child undernutrition was defined along three anthropometric indices: underweight, stunting and wasting. Weight measurements were obtained using lightweight, SECA mother-infant scales with a digital screen, designed and manufactured under the guidance of UNICEF. Height measurements were carried out using a measuring board manufactured by Shorr Productions. Children younger than 24 months were measured for height while lying down, and older children, while standing [6]. The WHO 2006 growth standards were used to transform children’s weight and length/height measurements into sex- and age-specific Z-scores: height-for-age Z-score (HAZ), weight-for-age Z-score (WAZ) and weight-for-height Z-score (WHZ) [7]. Stunting was defined as HAZ below -2SD, underweight was defined as WAZ below -2SD while wasting was defined as WHZ below -2SD from the respective WHO 2006 growth standards reference median.

#### Exposure variables

The nutritional status of women was assessed by use of height and body mass index (BMI). To derive BMI, EDHS measured the height and weight of women age 15–49 years. BMI is used to measure thinness or obesity. BMI is defined as weight in kilograms divided by height in meters squared (kg/m^2^). A BMI below 18.5 kg/m^2^ indicates thinness or acute undernutrition. A BMI below 17 kg/m^2^ indicates severe undernutrition. A BMI of 25.0 kg/m^2^ or above indicates overweight or obesity. Height was also classified in a single cut off point < 145 cm as short stature.

### Covariates

We included a number of theoretically important covariates that have been considered before in other studies on childhood undernutrition [8]. Child’s sex and age, maternal age, maternal education, place of residence (urban and rural). We also included a number of additional covariates: maternal smoking status, maternal parity and household wealth index.

### Data analysis

DHS has developed recode files in order to facilitate data analysis. Recode files have standard data definitions across countries and across DHS phases. There are seven common types of recode data files associated with the core questionnaires. The datasets are available in the standard recode file formats in SPSS, SAS, Stata and CSPro; only completed questionnaires are included in these files. Among the types of the recode data files, children recode file is one of them. This is a dataset that has one record for every child of interviewed women, born in the five years preceding the survey. Therefore, we used the children recode data file in the form of Stata for analysis.

**Fig. 1:**
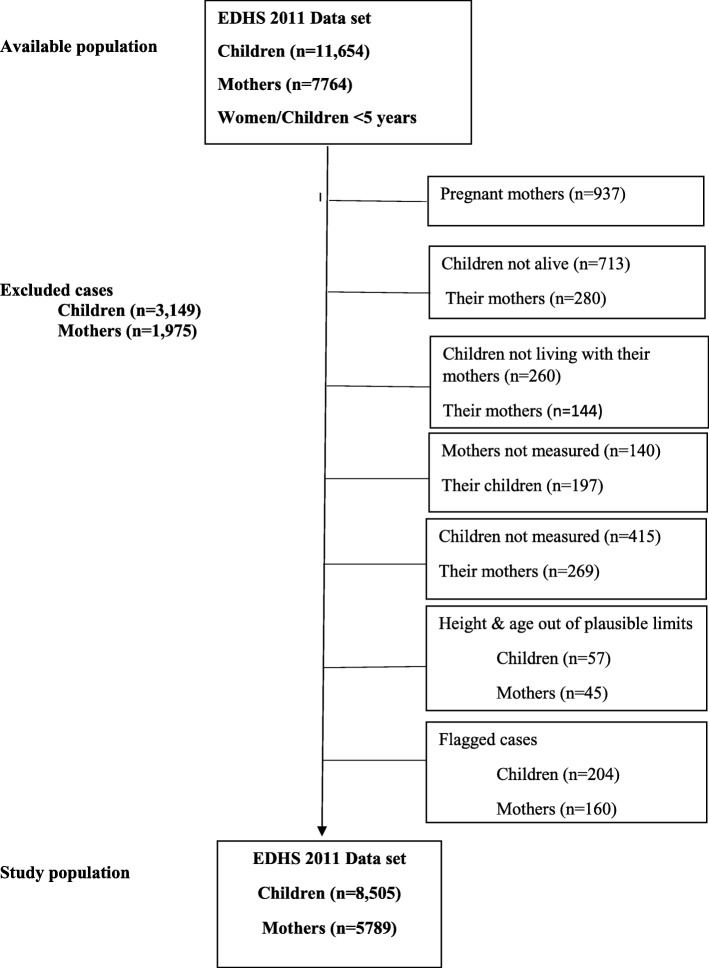
Study population diagram

The available sample in the child recode file was 11, 654 children under age five and 7764 mothers. Of the 11,654 children, we excluded from the analysis children from pregnant mothers (1303); children not alive at the time of interview (*n* = 713); children not living with their mothers (*n* = 260); children not measured (*n* = 612); and values that are flagged and out the plausible limit (*n* = 261). The final data set comprised 8505 children aged 0–59 months (Fig. 1). We have not used child-mother pairs instead we have used all children and repeated their mothers. For example, if the mother had three children, she was repeated three times. It’s not recommended to match mother with the younger child by doing this we will introduce bias because the youngest child born in the last five years tend to be healthier than other children [9]. It would have been better to select the matched child at random but that means it’s impossible to match our results exactly. Statistical analysis was performed using the STATA software package, version 14.0 (Stata Corp., College Station, TX, USA). Its survey commands (*svy*) account for the complex sample survey data composition: strata, clusters, and weights.

We estimated weighted prevalence of stunting, underweight and wasting by maternal, child and household variables. Overall differences across the categories were tested with design-based Pearson chi-squared test. We also carried out correlation analysis to investigate possible associations between nutritional indices of the mother and the children and statistical significance was considered at the significance level of 5%. STATA does not give confidence intervals for correlations, so new command (*corrci*) was used to estimate confidence intervals. We then calculated sensitivity, specificity, predictive values and Area Under Roc Curve of the nutrition indices of the mother and the children using *diagt* command. Maternal BMI and height was used to predict the nutrition status of the children. The area under the ROC curve (AUC) determines the overall level of accuracy, with a value of 0.50 indicating purely random performance and 1.00 indicating the maximal value possible. According to an arbitrary guideline, one could distinguish between non-informative (AUC = 0.5), less accurate (0.5 < AUC ≤0.7), moderately accurate (0.7 < AUC ≤ 0.9), highly accurate (0.9 < AUC < 1) and perfect tests (AUC = 1, [10]).

### Regression analysis

In evaluating the predictors of child undernutrition, logistics regression for binary outcomes was used. Unadjusted and adjusted odd ratios from logistic regression with corresponding 95% confidence interval was used to assess the significance and the magnitude of the effects of the given exposure.

## Result

### Background characteristics

The background characteristics of children are presented in Table 1. Fifty-one percent of children were male and the rest were females. Twenty-three percent of children were aged 0–11 months and 21% were aged 36–47 months. Of all children, 42.9% were stunted, 28.2% were underweight, and 10% exhibited wasting. Mean maternal BMI of stunted children was 20.01 (95% CI: 19.86–20.16), whereas that for children who were not stunted was 20.46 (95% CI, 20.31–20.62). Similarly, children who experienced underweight or wasting had mothers who had consistently lower BMI than those who did not (*p* < 0.001). Lower prevalence of child undernutrition was observed as the maternal BMI increases. The same is true for maternal height; children who experienced undernutrition had shorter mothers except for wasting (*p* < 0.05). We also examined the distribution of covariates across categories of child undernutrition. Higher prevalence of undernutrition was also observed among children of male sex and older ages and among children whose mothers have lower levels of education and among children from rural area and poorest households (*p* < 0.05). We have found no relationship between maternal parity with underweight and wasting. Likewise, no relationship was found between maternal age, marital status and smoking status with the three forms of child undernutrition.

**Table 1:** Frequency and Weighted Prevalence of Stunting, Underweight and Wasting of Children age less than 5 Years According to Maternal Anthropometry and Other Maternal, Child, and Household Characteristics. EDHS 2011

	< − 2 SD^1^						
		*p* ^*2*^		*p* ^*2*^		*p* ^*2*^	
Variable	HAZ^3^		WAZ^4^		WHZ^5^		Total
Total n (weighted %)	3478 (42.9)		2472 (28.2)		1007 (10.01)		8505 (100)
Child’s sex n (weighted %)		0.03		0.001		0.001	
M	1824 (44.7)		1337 (30.4)		575 (11.5)		4312 (51)
F	1654 (41.1)		1135 (25.9)		432 (8.5)		4193 (49)
Child’s age in month n (weighted %)		< 0.001		< 0.001		< 0.001	
0–11	267 (15.6)		319 (15.3)		331 (14.7)		1943 (22.9)
12–23	699 (44.8)		500 (29.6)		255 (13.5)		1612 (18.6)
24–35	817 (55.8)		542 (33.9)		145 (8.8)		1515 (17.5)
36–47	931 (55.3)		584 (32.9)		134 (6.1)		1766 (21.2)
48–59	764 (48.2)		527 (31.6)		142 (6.6)		1669 (19.8)
Maternal BMI n (weighted %)		< 0.001		< 0.001		< 0.001	
< 17 kg/m^2^	317 (42.5)		302 (41)		158 (16.9)		760 (5.9)
17.0–18.4 kg/m^2^	738 (47.9)		614 (37.6)		260 (13.4)		1622 (16.8)
18.5–24.9 kg/m^2^	2314 (43.0)		1505 (25.9)		563 (8.9)		5646 (73.3)
≥ 25 kg/m^2^	109 (22.3)		51 (11.7)		26 (7.2)		477 (4.1)
Maternal height n (weighted %)		< 0.001		0.01		0.93	
< 145 cm	108 (61.3)		77 (39.4)		22 (10.3)		177 (2.2)
≥ 145 cm	3370 (42.5)		2395 (27.9)		985 (10.0)		8328 (97.8)
Maternal age n (weighted %)		0.18		0.14		0.54	
15–19	108 (36.8)		84 (25.4)		49 (12.9)		357 (3.9)
20–24	639 (40.7)		436 (26.1)		199 (10.5)		1675 (20.0)
25–29	1069 (43.0)		757 (28.8)		300 (10.2)		2638 (31.4)
30–34	738 (44.9)		542 (30.2)		214 (9.0)		1726 (19.6)
35–39	575 (42.4)		399 (25.8)		148 (9.3)		1348 (15.6)
40–44	274 (48.3)		207 (33.7)		81 (11.3)		571 (7.1)
45–49	75 (42)		47 (25.1)		16 (7.0)		190 (2.4)
Maternal education in years n (weighted %)		< 0.001		< 0.001		0.002	
No education	2606 (45.3)		1921 (30.6)		770 (11.1)		5897 (69.3)
Primary	790 (40.3)		513 (24.6)		2151 (8.1)		12,174 (25.6)
Secondary	62 (19.3)		32 (11.4)		14 (3.1)		289 (3.4)
Higher	20 (18)		6 (4.8)		18 (5.6)		145 (1.7)
Maternal parity n (weighted %)^6^		0.003		0.0.09		0.0.40	
1	1354 (44.0)		921 (28.6)		366 (9.6)		3242 (38.1)
2	1703 (44.0)		1241 (29.0)		516 (10.6)		4109 (48.3)
≥ 3	421 (35.7)		310 (23.8)		125 (9.0)		1154 (13.6)
Maternal marital status n (weighted %)		0.37		0.15		0.07	
Never in union	19 (49.3)		10 (22.3)		6 (9.6)		51 (0.6)
Married	3044 (42.7)		2195 (28.6)		901 (10.4)		7448 (87.6)
Living with partner	146 (40.2)		93 (23.8)		35 (5.8)		424 (5.0)
Widowed	77 (45.4)		44 (18.5)		15 (4.0)		165 (1.9)
Divorced	142 (49.8)		100 (30.8)		35 (10.1)		286 (3.4)
Separated	50 (46.3)		30 (28.5)		15 (7.5)		131 (1.5)
Maternal smoking status n (weighted %)		0.06		0.46		0.85	
No	3452 (42.9)		2453 (28.3)		1001 (10.0)		8447 (99.7)
Yes	26 (64.7)		19 (36.5)		9 (11.3)		58 (0.3)
Place of residence n (weighted %)		< 0.001		0.0002		< 0.001	
Urban	371 (31.4)		221 (16.9)		115 (5.5)		1401 (13)
Rural	3107 (44.6)		2251 (29.9)		892 (10.7)		7104 (87)
Household wealth index n (weighted %)		< 0.001		< 0.001		< 0.001	
Poorest	1145 (47.3)		925 (35.3)		415 (13.4)		2572 (22.4)
Poor	738 (46.0)		531 (32.1)		191 (12.8)		1569 (21.9)
Middle	611 (44.8)		356 (25.6)		160 (9.1)		1424 (20.8)
Richer	583 (43.4)		356 (25.7)		135 (7.8)		1410 (19.9)
Richest	401 (28.3)		223 (14.4)		106 (5.2)		1530 (15.0)
Maternal BMI, mean (SE)^7^, kg/m^2^	20.01 (0.08)	< 0.001^8^	19.7 (0.10)	< 0.001^8^	19.7 (0.22)	< 0.001^8^	20.3 (0.06)
Maternal height, mean (SE), cm	155.4 (0.17)	< 0.001	155.6 (0.20)	< 0.001	156.2 (0.34)	0.74	156.6 (0.14)
Child age, mean (SE), months	33.9 (0.37)	< 0.001	32.5 (0.45)	< 0.001	22.9 (0.82)	< 0.001	28.9 (0.26)

^1^SD: Standard Deviation, ^2^Design-based Pearson chi-squared test, ^3^HAZ: height-for-age z-score, ^4^WAZ: weight-for-age z-score

^5^WHZ: weight-for-height z-score, ^6^Total number of births in the last five years, ^7^Mean and Standard Error accounting for complex design

^8^t-test (mean comparison test)

### Correlations between nutritional status of mothers and their children

The result on the correlation between maternal and children nutritional indicators is presented in Table 2. Maternal and child nutritional status were positively correlated at statistically significant levels. However, the strength of association was low and varied considerably across child age groups. For the overall sample, only WAZ appear to be more strongly correlated with maternal weight (r = 0.212, *p* < 0.05) and BMI (r = 0.281, *p* < 0.05) than HAZ and WHZ. In addition, HAZ is more strongly correlated with maternal height (r = 0.192, *p* < 0.05) than WAZ and WHZ. The age-disaggregated data revealed considerable variability in the strength of the correlations. The correlations between maternal and child nutrition levels were higher among 12–23 months old than the rest of the age groups. In the remaining age groups, the correlation patterns across age groups vary, depending on the index. Overall, WAZ was consistently positively correlated with maternal nutritional indices.

**Table 2:** Correlation coefficients (95% CI) between Maternal and Child Anthropometry Indices. EDHS, 2011

	Maternal Anthropometry (Pearson correlation coefficients)		
Child anthropometry	BMI	Weight	Height
0–59 months (*n* = 8505)			
^1^HAZ	0.109 (0.088, 0.130) *	0.203 (0.183, 0.224) *	0.192 (0.171, 0.212) *
^2^WAZ	0.212 (0.192, 0.232) *	0.281 (0.261, 0.301) *	0.141 (0.120, 0.161) *
^3^WHZ	0.191 (0.170, 0.211) *	0.178 (0.157, 0.198) *	− 0.026(− 0.047, − 0.005)
0–11 months (*n* = 1943)			
HAZ	0.099 (0.055, 0.143) *	0.229 (0.186, 0.270) *	0.261 (0.219, 0.302) *
WAZ	0.201 (0.158, 0.244) *	0.281 (0.240, 0.322) *	0.193 (0.150, 0.236) *
WHZ	0.163 (0.119, 0.206) *	0.123 (0.079, 0.167) *	− 0.037(− 0.081, 0.008)
12–23 months (*n* = 1612)			
HAZ	0.126 (0.078, 0.174) *	0.232 (0.185, 0.277) *	0.214 (0.167, 0.260) *
WAZ	0.250 (0.204, 0.295) *	0.331 (0.287, 0.374) *	0.151 (0.103, 0.199) *
WHZ	0.204 (0.156, 0.250) *	0.254 (0.208, 0.300) *	0.015(− 0.034, 0.064)
24–35 months (*n* = 1515)			
HAZ	0.137 (0.088, 0.187) *	0.205 (0.156, 0.253) *	0.153 (0.103, 0.053)
WAZ	0.201 (0.152, 0.203) *	0.251 (0.203, 0.298) *	0.103 (0.053, 0.153) *
WHZ	0.155 (0.105, 0.203) *	0.156 (0.106, 0.205) *	− 0.010(− 0.061, 0.040)
36–47 months (*n* = 1766)			
HAZ	0.092 (0.046, 0.138) *	0.195 (0.150, 0.240) *	0.191 (0.146, 0.236) *
WAZ	0.179 (0.134, 0.224) *	0.271 (0.227, 0.314) *	0.127 (0.081, 0.173) *
WHZ	0.166 (0.120, 0.211) *	0.182 (0.137, 0.227) *	− 0.036(− 0.082, 0.011)
48–59 months (*n* = 1669)			
HAZ	0.099 (0.051, 0.146) *	0.200 (0.153, 0.246) *	0.228 (0.182, 0.273) *
WAZ	0.250 (0.204, 0.294) *	0.294 (0.250, 0.338) *	0.142 (0.095, 0.189) *
WHZ	0.258 (0.212, 0.302) *	0.197 (0.151, 0.243) *	− 0.072(− 0.119, − 0.024)

^1^HAZ: height-for-age z-score, ^2^WAZ: weight-for-age z-score, ^3^WHZ: weight-for-height z-score

*P < 0.05

### Screening for child undernutrition based on maternal BMI

The result on the use of maternal nutritional status as a screening tool for child undernutrition is presented in Tables 3 and 4. For the overall sample of children, the sensitivity of maternal underweight (defined by BMI < 18.5 kg/m^2^) as a predictor of child nutritional status (<− 2 z-scores) is low, failing to reach 50% for any of the child nutrition indices. WHZ performed better than the other indicators for the full sample of children. Conversely, specificity was generally quite high (> 73). The positive predictive value of the screening was also low (< 44%), irrespective of the anthropometric indicator considered while the negative predictive values are fairly high (> 74%) with an exception of HAZ. With the age-disaggregated data, sensitivity tends to improve as the age of child increases for all child anthropometric indices with WHZ reaching 50.2%. However, this incremental pattern does not appear to go beyond the age of 23 months. The patterns vary across the rest of age groups depending on the index. Overall, maternal BMI (< 18.5 kg/m^2^) was less accurate but relatively better predictor of wasting than underweight and stunting AUC = 0.577(0.561, 0.593).

**Table 3:** Result of screening for child undernutrition based on maternal BMI ≤18.5 kg/m^2^. EDHS, 2011

	95% Confidence Interval				
Anthropometric indices	Sensitivity	Specificity	PPV^1^	NPV^2^	^3^AUC
0–59 months (*n* = 8505)					
^4^HAZ (< − 2)	30.3 (28.8, 31.9)	73.6 (72.4, 74.9)	44.3 (42.3, 46.3)	60.4 (59.2, 61.7)	0.52 (0.51, 0.529)
^5^WAZ (< −2)	37.1 (35.1, 39)	75.7 (74.6, 76.8)	38.5 (36.5, 40.4)	74.6 (73.5, 75.7)	0.564 (0.553, 0.575)
^6^WHZ (< −2)	41.5 (38.4, 44.6)	73.8 (72.8, 74.8)	17.5 (16, 19.1)	90.4 (89.6, 91.1)	0.577 (0.561, 0.593)
0–11 months (*n* = 1943)					
HAZ (< −2)	29.2 (23.8, 35.1)	76.3 (74.2, 78.3)	16.4 (13.2, 20.1)	87.1 (85.3, 88.8)	0.528 (0.498, 0.557)
WAZ (< −2)	31 (26, 36.4)	76.8 (74.7, 78.9)	20.8 (17.3, 24.8)	85 (83.1, 86.8)	0.539 (0.512, 0.567)
WHZ (< −2)	32 (27, 37.3)	77.1 (75, 79.1)	22.3 (18.6, 26.3)	84.7 (82.7, 86.5)	0.546 (0.518, 0.573)
12–23 months (n = 1612)					
HAZ (< −2)	34.5 (31, 38.1)	70 (66.9, 72.9)	46.8 (42.4, 51.2)	58.2 (55.3, 61.2)	0.522 (0.499, 0.545)
WAZ (< −2)	43.6 (39.2, 48.1)	73.3 (70.6, 75.9)	42.3 (38, 46.7)	74.3 (71.6, 76.9)	0.584 (0.559, 0.61)
WHZ (< − 2)	50.2 (43.9, 56.5)	71.5 (69, 73.9)	24.9 (21.2, 28.8)	88.4 (86.4, 90.3)	0.608 (0.575, 0.641)
24–35 months (n = 1515)					
HAZ (< − 2)	29.9 (26.7, 33.1)	73.8 (70.4, 77)	57.1 (52.3,61.9)	47.3 (44.3, 50.4)	0.518 (0.496, 0.541)
WAZ (< −2)	37.1 (33, 41.3)	76.8 (74, 79.4)	47.1 (42.3, 51.9)	68.7 (65.8, 71.4)	0.569 (0.545, 0.594)
WHZ (< −2)	37.9 (30, 46.4)	72.8 (70.4, 75.2)	12.9 (9.85, 16.4)	91.7 (89.9, 93.3)	0.554 (0.513, 0.595)
36–47 months (n = 1766)					
HAZ (< −2)	28.6 (25.7, 31.6)	72.6 (69.4, 75.6)	53.7 (49.2, 58.2)	47.7 (44.9, 50.5)	0.506 (0.485, 0.527)
WAZ (< −2)	34.1 (30.2, 38.1)	75 (72.4, 77.4)	40.2 (35.9, 44.9)	69.7 (67.1, 72.2)	0.545 (0.552, 0.568)
WHZ (< −2)	44.8 (36.2, 53.6)	73.3 (71.1, 75.5)	12.1 (9.38, 15.3)	94.2 (92.7, 95.4)	0.591 (0.547, 0.634)
48–59 months (n = 1669)					
HAZ (< − 2)	29.6 (26.4, 33)	73 (70, 75.9)	48.1 (43.5, 52.7)	55.1 (52.3, 58)	0.513 (0.491, 0.535)
WAZ (< −2)	37.8 (33.6, 42.1)	76.3 (73.7, 78.7)	42.3 (37.8, 47)	72.6 (70, 75.2)	0.57 (0.546, 0.594)
WHZ (< −2)	48.6 (40.1, 57.1)	73.7 (71.5, 75.9)	14.7 (11.6, 18.2)	93.9 (92.4, 95.2)	0.612 (0.569, 0.654)

^1^PPV: Positive predictive value, ^2^NNP: Negative predictive value, ^3^AUC: Area Under Roc Curve, ^4^HAZ: height-for-age z-score

^5^WAZ: weight-for-age z-score, ^6^WHZ: weight-for-height z-score

**Table 4:** Result of screening for child undernutrition based on maternal nutritional status (Height < 145 cm). EDHS, 2011

	95% Confidence Interval				
Anthropometric indices	Sensitivity	Specificity	^1^PPV	^2^NPV	^3^AUC
0–59 months (n = 8505)					
^4^HAZ (< −2)	3.11 (2.55, 3.74)	98.6 (98.3, 98.9)	61 (53.4, 68.2)	59.5 (58.5, 60.6)	0.509 (0.505, 0.512)
^5^WAZ (< −2)	3.11 (2.47, 3.88)	98.3 (98, 98.6)	43.5 (36.1, 51.1)	71.2 (70.3, 72.2)	0.507 (0.504, 0.511)
^6^WHZ (< −2)	2.18 (1.37, 3.29)	97.9 (97.6, 98.2)	12.4 (7.96, 18.2)	88.2 (87.5, 88.9)	0.501 (0.496, 0.505)
0–11 months (n = 1943)					
HAZ (< −2)	6.37 (3.75, 10)	98.5 (97.8, 99)	40.5 (25.6, 56.7)	86.8 (85.2, 88.3)	0.524 (0.509, 0.539)
WAZ (< −2)	5.96 (3.96, 9.15)	98.6 (97.9, 99.1)	45.2 (29.8, 62.3)	84.2 (82.5, 85.8)	0.523 (0.509, 0.536)
WHZ (< −2)	2.11 (0.85, 4.31)	97.8 (97, 98.5)	16.7 (6.79, 31.4)	83 (81.2, 84.6)	0.5 (0.491, 0.508)
12–23 months (n = 1612)					
HAZ (< −2)	3.43 (2.21, 5.07)	98.4 (97.3, 99.1)	61.5 (44.6, 76.6)	57.1 (54.6, 59.6)	0.509 (0.501, 0.517)
WAZ (< −2)	3.6 (2.15 (5.63)	98.1 (97.1, 98.8)	46.2 (30.1, 62.8)	69.4 (67, 71.6)	0.509 (0.499, 0.518)
WHZ (< −2)	3.53 (1.63, 6.59)	97.8 (96.9, 98.5)	23.1 (11.1, 39.3)	84.4 (82.5, 86.1)	0.507 (0.495, 0.519)
24–35 months (n = 1515)					
HAZ (< −2)	1.96 (1.12, 3.16)	99.3 (98.3, 99.8)	76.2 (52.8, 91.8)	46.4 (43.8, 49)	0.506 (0.501, 0.512)
WAZ (< −2)	1.85 (0.89, 3.37)	99.9 (98, 99.4)	47.6 (25.7, 70.2)	64.4 (61.9, 66.8)	0.504 (0.497, 0.51)
WHZ (< −2)	0.69 (0.18, 3.78)	98.5 (97.8, 99.1)	4.76 (0.12, 23.8)	90.4 (88.8, 91.8)	0.496 (0.489, 0.504)
36–47 months (n = 1766)					
HAZ (< −2)	2.9 (1.92, 4.19)	98.3 (97.2, 99.1)	65.9 (49.4, 79.9)	47.6 (45.2, 50)	0.506 (0.499, 0.513)
WAZ (< −2)	2.23 (1.19, 3.78)	97.6 (96.6, 98.4)	31.7 (18.1, 48.1)	66.9 (64.6, 69.1)	0.499 (0.492, 0.507)
WHZ (< −2)	2.24 (0.46, 6.4)	97.7 (96.8, 98.3)	7.32 (1.54, 19.9)	92.4 (91.1, 93.6)	0.5 (0.486, 0.513)
48–59 months (n = 1669)					
HAZ (< −2)	3.14 (2.02, 4.64)	98.9 (98, 99.5)	70.6 (52.5, 84.9)	54.7 (52.3, 57.2)	0.51 (0.503, 0.517)
WAZ (< −2)	3.23 (1.89, 5.11)	98.5 (97.6, 99.1)	50 (32.4, 67.6)	68.8 (66.5, 71)	0.509 (0.5, 0.57)
WHZ (< −2)	1.41 (0.17, 5)	97.9 (97.1, 98.6)	5.88 (0.72, 19.7)	91.4 (90, 92.7)	0.497 (0.486, 0.507)

^1^PPV: Positive predictive value, ^2^NNP: Negative predictive value, ^3^AUC: Area Under Roc Curve

^4^HAZ: height-for-age z-score, ^5^WAZ: weight-for-age z-score, ^6^WHZ: weight-for-height z-score

### Screening for child undernutrition based on maternal height

As indicated in Table 4, the use of maternal height (defined by height < 145 cm) as a screening tool for child malnutrition had even lower sensitivity (< 4% for all anthropometric indicators) than for screening performed using maternal BMI, though the specificity was higher (> 98%). Disaggregating the data by age of the child did not alter the overall conclusions.

### Logistics regression

The multivariate model presented in Table 5 indicated that child’s age and sex, maternal BMI, place of residence and household wealth index were significantly associated with the three forms of child’s undernutrition. Maternal height and maternal education were significantly associated with child’s stunting and underweight.

**Table 5:** Predicators of child undernutrition, EDHS 2011

Variables	Stunting		Underweight		Wasting	
	Crude OR (95% CI)	Adjusted OR (95% CI)	Crude OR (95% CI)	Adjusted OR (95% CI)	Crude OR (95% CI)	Adjusted OR (95% CI)
Child’s sex						
Male	1.13 (1.03, 1.23) *	1.14 (1.04, 1.26) *	1.21 (1.10, 1.33) **	1.23 (1.12, 1.36) **	1.34 (1.17, 1.53) **	1.36 (1.18, 1.55) **
Female	1.00	1.00	1.00	1.00	1.00	1.00
Child’s age in months						
0–11	1.00	1.00	1.00	1.00	1.00	1.00
12–23	4.81 (4.09, 5.65) **	4.93 (4.18, 5.82) **	2.28 (1.95, 2.69) **	2.28 (1.93, 2.69) **	0.92 (0.77, 1.09)	0.87 (0.72, 1.04)
24–35	7.35 (6.24, 8.66) **	7.83 (6.61, 9.27) **	2.84 (2.42, 3.33) **	2.97 (2.52, 3.51) **	0.52 (0.42, 0.63) **	0.50 (0.40, 0.62) **
36–47	7.00 (5.97, 8.21) **	7.10 (6.03, 8.36) **	2.52 (2.15, 2.94) **	2.46 (2.10, 2.89) **	0.40 (0.32, 0.49) **	0.37 (0.30, 0.46) **
48–59	5.30 (4.51, 6.23) **	5.51 (4.67, 6.50) **	2.35 (2.01, 2.75) **	2.33 (1.98, 2.75) **	0.45 (0.37, 0.56) **	0.42 (0.34, 0.52) **
Maternal BMI						
< 18.5 kg/m2	1.21 (1.10, 1.34) **	1.09 (0.98, 1.21)	1.83 (1.66, 2.03) **	1.68 (1.52, 1.87) **	2.00 (1.75, 2.29) **	1.90 (1.65, 2.19) **
≥ 18.5 kg/m2	1.00	1.00	1.00	1.00	1.00	1.00
Maternal height						
< 145 cm	2.30 (1.70, 3.12) **	2.52 (1.81, 3.51) **	1.91 (1.41, 2.58) **	1.99 (1.45, 2.72) **	1.06 (0.67, 1.66)	1.07 (0.67, 1.69)
≥ 145 cm	1.00	1.00	1.00	1.00	1.00	1.00
Maternal age						
15–19	1.00	1.00	1.00	1.00	1.00	1.00
20–24	1.42 (1.11, 1.82) *	1.23 (0.94, 1.61)	1.14 (0.87, 1.49)	1.11 (0.84, 1.47)	0.85 (0.61, 1.19)	1.07 (0.75, 1.51)
25–29	1.57 (1.24, 1.99) **	1.22 (0.94, 1.60)	1.31 (1.01, 1.69) *	1.19 (0.90, 1.57)	0.81 (0.58, 1.12)	1.07 (0.76, 1.50)
30–34 35–39	1.72 (1.35, 2.20) ** 1.71 (1.33, 2.20) **	1.26 (0.96, 1.65) 1.16 (0.88, 1.53)	1.49 (1.14, 1.94) * 1.37 (1.04, 1.79) *	1.25 (0.94, 1.65) 1.09 (0.82,1.46)	0.89 (0.64, 1.24) 0.78 (0.55, 1.10)	1.12 (0.79, 1.60) 1.01 (0.70, 1.45)
40–44	2.12 (1.61, 2.81) **	1.27 (0.93, 1.73)	1.85 (1.37, 2.49) **	1.36 (0.99, 1.87) *	1.04 (0.71, 1.52)	1.40 (0.94, 2.09)
45–49	1.50 (1.04, 2.17) *	0.78 (0.53, 1.15)	1.07 (0.71, 1.61)	0.71 (0.46, 1.08)	0.58 (0.32, 1.05)	0.79 (0.43, 1.46)
Maternal education						
No education	4.95 (3.08, 7.96) **	3.00 (1.82, 4.95) **	11.1 (4.93, 25.4) **	5.33 (2.31, 12.3) **	2.57 (1.26, 5.27) *	1.62 (0.76, 3.45)
Primary	3.57 (2.21, 5.77) **	2.58 (1.56, 4.26) **	7.15 (3.14, 16.3) **	4.20 (1.82, 9.68) *	1.88 (0.91, 3.89)	1.27 (0.60, 2.69)
Secondary	1.71 (0.99, 2.96)	1.82 (1.03, 3.19) *	2.88 (1.18, 7.07) *	2.62 (1.06, 6.48) *	0.87 (0.36, 2.13)	0.68 (0.28, 1.70)
Higher	1.00	1.00	1.00	1.00	1.00	1.00
Maternal parity						
1	1.00	1.00	1.00	1.00	1.00	1.00
2	0.99 (0.90, 1.08)	0.91 (0.82, 1.01) *	1.09 (0.99, 1.21)	0.96 (0.86, 1.07)	1.13 (0.98, 1.30)	1.00 (0.86, 1.16)
≥ 3	0.80 (0.70, .92) *	0.74 (0.63 0.86) **	0.93 (0.80, 1.08)	0.84 (0.72, 0.99) *	0.95 (0.77, 1.18)	0.90 (0.72, 1.13)
Place of residence						
Urban	1.00	1.00	1.00	1.00	1.00	1.00
Rural	2.16 (1.90, 2.45) **	1.37 (1.12, 1.68) *	2.48 (2.12,2.88) **	1.20 (0.95, 1.51)	1.61 (1.31, 1.97) **	0.82 (0.60, 1.11)
Household wealth index						
Poorest	2.26 (1.97, 2.59) **	1.70 (1.38, 2.10) **	3.29 (2.80, 3.88) **	2.23 (1.76, 2.82) **	2.58 (2.07, 3.23) **	2.23 (1.60, 3.10) **
Poor	2.50 (2.15, 2.91) **	1.89 (1.51, 2.35) **	3.00 (2.51, 3.58) **	2.10 (1.64,2.69) **	1.86 (1.45, 2.39) **	1.72 (1.21, 2.43) *
Middle	2.12 (1.81, 2.47) **	1.59 (1.28, 1.99) **	2.59 (2.16, 3.11) **	1.86 (1.45, 2.39) **	1.70 (1.32, 2.20) **	1.60 (1.12, 2.28) *
Richer	1.98 (1.70, 2.32) **	1.45 (1.17, 1.80) **	1.98 (1.64, 2.38) **	1.42 (1.12, 1.81) *	1.42 (1.09, 1.85) **	1.39 (0.98, 1.95)
Richest	1.00	1.00	1.00	1.00	1.00	1.00

*P < 0.05

**P < 0.001

Male gender was associated with stunting (AOR 1.23; 95% CI; 1.12, 1.36), underweight (AOR 1.14; 95% CI; 1.04, 1.26) and wasting (AOR 1.36; 95% CI; 1.18, 1.55). The risk of undernutrition also varied by age groups. Children age 24–35 months were more likely to be stunted (AOR 7.86; 95% CI; 6.64, 9.20) and underweight (AOR 2.98; 95% CI; 2.53, 3.52). Underweight mothers were more likely to have stunted (AOR 1.09; 95% CI; 0.98, 1.21), underweight (AOR 1.68; 95% CI; 1.52, 1.87) and wasted (AOR 1.90; 95% CI; 1.65, 2.19) children. Moreover, children in the poorest households are more at risk of being malnourished compared to their counterparts in the richest household.

On the other hand, mothers whose height was < 145 cm tend to have stunted (AOR 2.50; 95% CI; 1.79, 3.49) and underweight (AOR 1.99; 95% CI; 1.45, 2.72) children. We did not find association between maternal height and child’s wasting. Likewise, children whose mothers had no education and reside in rural were more likely to be stunted and underweight.

## Discussion

Using a large and representative nationwide sample of children and mothers from Ethiopia, we aimed to examine if we can use maternal BMI and height as a proxy indicator of their children’s undernutrition and explore the predictors of child undernutrition. We observed a significant but weak correlation between mothers and their children’s nutritional status. We also found that child’s age and sex, maternal BMI, place of residence and household wealth index were significantly associated with the three forms of child’s undernutrition. Maternal height and maternal education were significantly associated with child’s stunting and underweight.

One of the main findings is the variability of the strength of correlation when the data was disaggregated according to the age of the child. The correlation between maternal and child nutrition status was lower for under 12 months and 24–35 months children than the older children for most of the indicators. This may be part as a result of increased measurement error among these age groups. However, since WAZ has no height measurement and tends to be less affected by field measurements than HAZ and WHZ, this suggest that the data have been affected by age variation among the under five children. Additionally, this study has shown that HAZ is more strongly correlated with maternal height than WAZ and WHZ. This result is expected since measures of cumulative exposure to socioeconomic and environmental risk factors should be more highly correlated than measures that respond to more immediate environmental change. The attained height of the adult is a consequence of genetic and environmental effect on the growing period of childhood [11]. For instance, the stunted mother may provide an inadequate supply of nutrients for the fetus, which restricts fetal growth and promotes low birth weight and stunting in the children [12–14].

The low sensitivity of the maternal undernutrition as screening test suggests that factors associated with undernutrition tend not to be fully shared by mothers and their off springs. On the other hand, the high specificity suggests that factors which mitigate nutritional risk tend to be fully shared by mothers and their children. In other words, when mothers are well nourished their children tend also to be well nourished. Likewise, the low PPV and the high NPV suggest that knowledge about mother undernutrition only slightly improves the likelihood of correctly classifying the child as being malnourished. Our results are analogous with studies done in Guinea [15] and Ethiopia [16].

The following factors may explain the lack of stronger correlation between mothers and their children: First, the causes of undernutrition are multidimensional and they include both food and non-food related factors that often interact to form a complex web. Social, political, economic and environmental factors by large cause climate change resulting in variation in food supply [17]. Second, the relationship between maternal and child could be also mediated by the dual burden of malnutrition. Even in food secure households some members may be undernourished while others may be overweight, suggesting that with the process of nutritional transition with economic development, it’s possible to see double burden households [18, 19]. This is even evident in our data; 22.3% of stunted, 11.7% of underweight and 7.2% of wasted children are from households with overweight mothers.

Third, using a single BMI cutoff still remain controversial [20]. A healthy BMI is likely to vary with age, sex, pregnancy and lactation, ethnicity and other factors [21]. Therefore, using a BMI < 18.5 kg/m^2^ as the cutoff for everyone and everywhere is likely to be too crude. There are also evidences that showed BMI is not sensitive enough to detect small but clinically significant weight [22].

Likewise, child undernutrition is mainly as a result of poor feeding practices and infection and unlike children, the predominant cause of reduced body weight in adults is reduced food intake to meet the energy requirements [23]. In nutshell, the above discussed factors may explain lack of stronger correlation between maternal undernutrition (BMI and height) and child undernutrition indicators.

The estimation of three forms of undernutrition (stunting, underweight and wasting) was comparable to that of EDHS report but we could not exactly replicate the figures except for wasting. We believe the main source of our discrepancy from the report is that we used the child recode file (KR file), whereas the DHS report uses the household recode file (PR file) for stunting, underweight, and wasting. Height and weight of all children under five and all women 15–49 are measured in the household interview. If the mother is alive and in the same household as the child, then the child will also be in the KR file. Otherwise, the child will not be in the KR file. In other words, the children who are in the PR file but not in the KR file are more likely to be stunted.

Age and gender are factors that influence children susceptibility to malnutrition. For that reason, our results show that the male children are underprivileged across all three forms of undernutrition. There studies that documented higher prevalence of undernutrition among male children compared to female children [11, 24–26]. These studies corroborate our findings and there could be a biological explanation. For instance, Wamani and his colleagues found from epidemiological and cohort studies that neonatal and preterm babies’ mortality and morbidity to be consistently higher in males and females in early life though the underlying mechanism is poorly understood. However, the reported male predominance suggests that boys are generally vulnerable [26]. The child’s age is also an important predictor for child undernutrition. We found undernutrition to be more prevalent in older than in younger children. Children among 24–35 months have the highest levels of undernutrition, and the youngest age group (0–11 months) has the lowest levels except for wasting and were significantly associated. The higher proportion of undernutrition among older children could be due to inappropriate child feeding practices [24].

Results on maternal education and place of residence and child undernutrition are similar with those of other studies [11, 24, 27–29]. Education and place residence are socio-economic indicators and are expected to reflect both current and ongoing access to resources. In our result, undernutrition is common among children from rural areas and whose mothers have lower levels of education and were significantly associated. Additionally, our data has shown that child undernutrition is more common among children in the poorest wealth quintiles than in the richest quintiles and almost two times more at risk of being undernourished than their richest counter parts. Our result is consistent with findings of studies that found that children in the poorest households are more at risk of being malnourished compared to their counterparts in the richest households [11, 24, 30]. This could be explained by the fact the rich are able to afford better living conditions that may improve child nutrition.

The following limitations need to be considered while interpreting the above findings. First, though the DHS data is of high quality, recall bias in reporting children birth histories remains a potential concern [31]. Moreover, a quality analysis of height data in 81 DHS surveys also showed clustering on whole and half centimeters, with some additional heaping at multiples of 5 and 10 cm [32]. Second, we have used all children and repeated their mothers and we did not match randomly a mother with her child. This is likely to bias our standard errors because we are not getting a full amount of new information by adding new children to the same mother. Instead we used the DHS clustering method (*svyset*) which accounted for this. The basic intuition is that, without DHS survey design, we would want to “cluster” on the household (all those with the same mother). But, since DHS requires us to cluster at the Primary Sampling Unit (PSU) level, and all children in a household are in the same PSU, we are already taking the within-household serial correlation in maternal BMI into account. Lastly, this study is limited by its cross-sectional nature and hence, we cannot determine the findings of the current study and elsewhere represent evidence of causal relationship between maternal nutrition status and child undernutrition. In addition, the direction of possible causality in maternal nutritional status and child undernutrition may not be the same across the life course.

Despite these limitations, our study has some persuasive strength. First, EDHS data are considered to be of high quality and reliable source of maternal and child health data. Second, the standardized procedures used by these surveys increases the quality of the data and also allows for comparability across countries. Third, it provides a nationally representative sample, allowing for drawing conclusions about the entire country. Moreover, we believe we used the appropriate statistical methods to investigate these relationships and controlling for important covariates.

## Conclusion

In conclusion, maternal BMI and height are significantly associated with the child undernutrition but not accurate proxy indicators of child undernutrition. Therefore, regardless of the specific explanation, our findings show that the sensitivity and specificity of maternal anthropometric indicators to identify growth deficits among children were too low to justify using maternal indicators as a replacement for child growth measurements.

## Abbreviations

AUC: Area Under Receiver Operating Curve
BAZ: Body Mass Index for Age Z-score
BMI: Body Mass Index
CI: Confidence Interval
DALYs: Disability Adjusted Life Years
EA: Enumeration Area
EDHS: Ethiopia Demographic and Health Survey
ETB: Ethiopian Birr
GNP: Gross National Product
HAZ: Height for Age Z-score
IRB: Institutional Review Board
MUAC: Mid Upper Arm Circumference
NRERC: National Research Ethics Review Committee
SGA: Small for Gestational Age
UNICEF: United Nations Children’s Fund
WAZ: Weight for Age Z-score
WHO: World Health Organization
WHZ: Weight for Height Z-score
